# Tracking the Spatial and Functional Gradient of Monocyte-To-Macrophage Differentiation in Inflamed Lung

**DOI:** 10.1371/journal.pone.0165064

**Published:** 2016-10-18

**Authors:** Debasish Sen, Stephen M. Jones, Erin M. Oswald, Henry Pinkard, Kaitlin Corbin, Matthew F. Krummel

**Affiliations:** Department of Pathology, University of California San Francisco, San Francisco, California, United States of America; "INSERM", FRANCE

## Abstract

Myeloid-derived cells such as monocytes, dendritic cells (DCs), and macrophages are at the heart of the immune effector function in an inflammatory response. But because of the lack of an efficient imaging system to trace these cells live during their migration and maturation in their native environment at sub-cellular resolution, our knowledge is limited to data available from specific time-points analyzed by flow cytometry, histology, genomics and other immunological methods. Here, we have developed a ratiometric imaging method for measuring monocyte maturation in inflamed mouse lungs *in situ* using real-time using 2-photon imaging and complementary methods. We visualized that while undifferentiated monocytes were predominantly found only in the vasculature, a semi-differentiated monocyte/macrophage population could enter the tissue and resembled more mature and differentiated populations by morphology and surface phenotype. As these cells entered and differentiated, they were already selectively localized near inflamed airways and their entry was associated with changes in motility and morphology. We were able to visualize these during the act of differentiation, a process that can be demonstrated in this way to be faster on a per-cell basis under inflammatory conditions. Finally, our *in situ* analyses demonstrated increases, in the differentiating cells, for both antigen uptake and the ability to mediate interactions with T cells. This work, while largely confirming proposed models for *in situ* differentiation, provides important *in situ* data on the coordinated site-specific recruitment and differentiation of these cells and helps elaborate the predominance of immune pathology at the airways. Our novel imaging technology to trace immunogenic cell maturation *in situ* will complement existing information available on *in situ* differentiation deduced from other immunological methods, and assist better understanding of the spatio-temporal cellular behavior during an inflammatory response.

## Introduction

The inflammation of an organ, as during allergy or infection, involves the recruitment of many cell types that are critical for local and systemic immune reactions. Key amongst these for prolonged inflammation are inflammatory monocytes, which can subsequently differentiate *in situ* into macrophages (MΦ) and inflammatory monocyte-derived dendritic cells (iDC or moDC, hereafter moDC)[1–3]. It is well recognized that monocytes and their offspring are key players in innate responses such as phagocytosis[4] and release of reactive oxygen species. Inflammatory DC are also critical players in driving local adaptive immune reactions by providing peptide-MHC complexes to incoming T cells [5].

In mice and human lungs, mature macrophages and monocyte-independent conventional CD11c^+^ DC (cDC) populations, including those subsets expressing CD11b/BDCA1 (mouse/human) or, CD103 or Xcr1/BDCA3 are considered to be the predominant lung-resident DCs under basal conditions [6,7]. However, monocytes and monocyte-derived cells, which bear the chemokine receptor *CX3CR1*, are thought to give rise to cells very closely resembling DCs under inflammatory conditions in mucosal organs and in Th2-type lung allergies in mice [7]. “Resident” monocytes, which may be subtly or partially differentiated, have also been suggested to patrol the lung in the absence of inflammation[3,8]. Finally, mononuclear cells from patients with bronchial asthma can be activated *in vitro* and *in vivo* to present antigens and may orchestrate immune reactions in mild asthma [9]. It is paramount to know how all of the lineages of monocytes relate to each other, how they populate tissue and exactly when they begin to be effective at acquiring antigens and engaging T cells.

Live-imaging represents a promising avenue to assess both function and differentiation within tissues and organs [10]. In our previous work, based on pulse-chase antigen uptake experiments, it was surmised that flow of APCs from parenchyma to airways was taking place [11] but the source of this pool was not clear. In general, though it is recognized that much of the APC population that populates the inflamed lung come from monocyte origin [7,8], the way in which those cells accumulate across the space of the lung and also differentiate in that same space is not well studied. Here, we sought to address how and where infiltrating *CX3CR1*^+^ monocytes differentiate into moDCs in normal and acute allergic conditions and progressively populate airway mucosa over the course of an allergic immune response. Our results support a model for airway disease, in which differentiating APC are focused upon the branching airways.

## Materials and Methods

### Mice

C57Bl/6 mice were purchased from Simonsen Laboratories. Transgenic mice where the *CX3CR1* gene was substituted by EGFP [12] (*CX3CR1*-EGFP mice) were purchased from Jackson Laboratories. Mice expressing mCherry under the CD11c promoter (CD11c^mCherry^ mice) were a kind gift of K. Khanna [13]. The *CX3CR1*-EGFP mice were bred to CD11c^mCherry^ mice to allow tracing progressive maturation of monocytes to DCs on a ratiometric Cherry:GFP color scale. Unless otherwise mentioned, progenies that were heterozygote for *CX3CR1*^-^EGFP and also expressed CD11c^mCherry^ (*CX3CR1*-EGFP x ^mCherry^CD11c mice) were used. All studies involving mice were specifically approved and performed under the strict guidance of by the Institutional animal care and use committee (IACUC) of UCSF (Approval Number: AN106779). Animals were housed in a barrier facility and maintained on standard chow. Animals under treatment were monitored daily for signs of pain or discomfort and euthanized when necessary.

### Induction of lung allergy in mice

Standard immunization protocols using ovalbumin or HDM as a model antigen was used, as previously described [11]. For the former, mice were injected intra-peritoneally (i.p.) with 100 μg of ovalbumin (Ova; Sigma-Aldrich) included in 100 μL of 1 mg/ml Alum adjuvant (Pierce) on days 0, 7 and 14. Subsequently, the mice were challenged with intranasal (i.n.) administrations of 100 μg Ova included in 40 μL of PBS (Invitrogen Life Technologies) on days 21, 22, 23. As control alum without Ova was administered i.p., followed by PBS alone via i.n. All analyses were performed on day 24 unless otherwise mentioned. Lungs were harvested and observed using vital two-photon (2P) microscopy or analyzed using flow-cytometry (see below). As a second model, house dust mite extract (HDM) based allergic model was used. Briefly, mice were immunized by i.n. doses of 100 μg HDM extract in PBS on days 0 and 7, and challenged with i.n. administration of 100 μg HDM extract in PBS on days 14–18. All analyses were performed on day 19. For tracing HDM granules in the lungs HDM extracts were labeled with 10 μM VPD450 (BD Biosciences) for 20 min at 37C followed by removal of excess VPD by dialysis using a 100,000 MW cutoff and subsequent filtering with 40 μm nylon mesh (BD Biosciences). Labeled HDM was administered on day 18 or the final challenge at ~100 μg in PBS.

### Lung leukocyte isolation and flow cytometry

Lung leukocytes were isolated and labeled for analyses by flow cytometry as previously described. Briefly, mice were euthanized using a lethal dose of 1.3% tribromoethanol (Avertin, Sigma-Aldrich) and exsanguinated from the descending aorta. Lung lobes were then isolated and placed in DMEM and were cut into small pieces with a sterile razor blade and digested at 37°C for 30 min in DMEM with 2% FCS, 400 U/ml collagenase D (Roche), and 0.25 mg/ml DNAse I (Roche). Digested tissue were then homogenized by pipetting, filtered through a 100 μm filter and stained for flow cytometry. Antibodies used included Siglec-F PE (E50-2440; BD Biosciences), Siglec-F Alexa Fluor 647 (E50-2440; BD Biosciences), Ly6C Alexa Fluor^™^ 647 (HK-14; eBioscience), Ly6G APC-Cy7 (1A8; Biolegend), CD45 Alexa Fluor^™^ 700 (30-F11; Biolegend), CD11b PerCP-Cy5.5 (M1-70; Biolegend), CD11c PE-Cy7 (N418; eBioscience), CD4 APC-Cy7 (GK1.5; Biolegend), MerTK Biotin (Polyclonal; R&D Systems), FcεRIα APC (MAR-1; eBioscience), CD64 PE (X54-5/7.1; Biolegend), MHC-II Alexa Fluor 488 (N22), MHC-II Biotin (N22), Streptavidin eFluor 450 (eBioscience), Streptavidin APC-Cy7 (Biolegend). Flow cytometry was performed on Fortessa (BD Biosciences) and analyzed using FlowJo (Tree Star).

### Vital labeling of cells in vasculature

For vital labeling of cells inside the vasculature a pulse-labeling method was used, as previously described [14]. Briefly, 2 μg of anti-mouse CD45 APC (Biolegend) included in 150 μL PBS was i.v. injected via tail vein for 5 min followed by immediate euthanasia using a lethal dose of 1.3% tribromoethanol (Avertin, Sigma-Aldrich). Control populations of neutrophils (all cells vascular) and alveolar macrophages (all cells extravascular) were monitored as controls.

### Vital labeling of monocytes in the blood

To label blood monocytes, 10^7^ 2 μm diameter coumarin-containing latex beads in about 100 μL PBS were injected i.v. [15] to saline or ova-treated mice at 48 h, 24 h and 1 h prior to harvesting lungs on day 24 of the asthma regime. Harvested cells were analyzed for bead contents by flow cytometry.

### Two photon imaging of viable lung slices

For real-time deep-tissue imaging, a custom-built resonant-scanning 2P microscope [16] equipped with 6 switchable photomultiplier tube detector (4 usable at one time), dual independently tunable lasers (5 W; 780–920 nm tunable MaiTai—Spectra-Physics and 10 W; 780–1050 nm tunable Chameleon—Coherent), allowing multi-color submicron-resolved fluorescent cell visualization at least up to 250 μm beneath the surface of the mounted tissue, was used. The lasers were individually tuned and maintained at a constant wavelength throughout the length of imaging. Automated XY movements of the stage (Prior), four-dimensional PMT acquisition board (Bitflow Raven), Piezo-electric depth controller (), electro-optic modulators for optimal laser power input to the specimen, were controlled using open-source Micromanager Software. A 25X 1.05 N.A. water-immersion objective (Olympus Ultra MPE) was used, and a spatial resolution of 0.4956 μm/pixel was achieved. Thus, each 480x400 XY plane spanned 238 μm x 198 μm. In most cases, multiple XY positions were acquired and tiled to compose large images of areas of interest in the specimen. An average of 10 video rate frames was used for image acquisition. Thick tissue sections of lungs were prepared as previously described [11,17]. Briefly, mice were euthanized using a lethal dose of 1.3% tribromoethanol (Avertin, Sigma-Aldrich) and exsanguinated. Exposed lungs were then inflated through the trachea with 37°C 1.5% low melting temperature Agarose (Fisher Scientific), and solidified with cold PBS (8–12°C). The left lobes were isolated, sliced into thick sections (300 μm) with a Vibratome^™^, mounted on plastic cover slips using Vetbond (3M) and then imaged on a temperature controlled stage (Warner Instruments) while in constant perfusion with oxygenated (95% O_2_; 5% CO_2_) RPMI without phenol red. For GFP the MaiTai laser was used, tuned to 900 nm, and emission band pass filter of 505/20 nm was used. For all other fluorophores, the Chameleon laser was used, tuned to 1030 nm. Emission band pass filters used for these fluorophores were: for second harmonics in the “violet-blue” channel, Coumarin beads, or VPD-450 (BD Biosciences) labeled cells 440/40 nm, for mCherry or CMTMR (Invitrogen) 575/50 nm, and for Alexa-fluor 647-labeled molecules 650/50 nm. Confocal imaging was performed on a Nikon A1R multiphoton confocal microscope equipped with a MaiTai DeepSee laser using a 20X 1.00 NA water-immersion objective (Olympus) at a spatial resolution of 1.13 μm/pixel, with each XY frame extending 512x512 pixels. For excitation, 405, 488 and 568 nm lasers were used. Emission band pass filters used were 450/50 for coumarin beads or VPD-450, 525/50 for GFP and 575/50 for mCherry. Images were analyzed using Imaris (Bitplane), Fiji (Open Source) and Matlab (Math Works) software.

### Fluorescence labeling of viable lung slices

Lung slices were prepared as previously described, after vibratome cutting lung slices were place in 500 μL RPMI supplemented with 10% normal rat serum. 0.5 μg of anti XCR1 Alexa 647 (Biolegend, USA) was added to the media. Slices were incubated for 45 min at 37°C. Slices were then washed 3X with PBS followed by transferring to ice could 10mL of RPMI with 10% FBS. Slices were imaged and analyzed as described above.

### Image Analysis

Using Imaris^™^, surfaces were generated based on Cherry or GFP signal intensity, using “Absolute Intensity”, a Surface area detail level of 2x the xy pixel size, and size cutoff of 200 μm^3^. Airway surfaces were manually traced in Imaris using the slice mode in the manual surface creation in Imaris, preserving surface features. Distance transform was used to generate a channel of intensities representing distances from the airway. Mean intensities of all channels, including the calculated distance channel was exported to Excel files. Ratio of Cherry to GFP was normalized by first calculating ratio = Mean intensity of Cherry/ mean intensity of GFP for each GFP+ surface (S3C Fig). Base 10 logarithmic values log(Ratio) of these ratios were then calculated. Finally the log(Ratio) values were normalized to a 0 to 100 scale, such that the minimum log(Ratio) value was scaled to 0 and the maximum log(Ratio) value was scaled to 100. “low”, “mid” and “hi” values were defined to be the zones in the lower, middle and upper thirds of this normalized scale, specifically 0–33, 34–66, 67–100. Additionally, AMs were removed or separately analyzed based on a combination of normalized Autofluorescence:GFP ratio and based on sphericity (S1B Fig), Cellular densities within distance bins were calculated as number of cells within 50 μm bins starting from the airway, divided by the total volume of space occupied by the bin in question. Tracks were calculated in Imaris and tracks were manually evaluated to prevent errors arising from automated tracking. Track displacements with respect to the airway were calculated using the distance to airway channel parameter from the track data. One-way ANOVA was performed to compute p-values between track speeds within “low”, “mid”, “hi” and Cherry^+^ only cells.

### Study Approval (Ethics)

This study was specifically approved by the IACUC of UCSF. APPROVAL NUMBER: AN106779

## Results

### A Ratiometric Color Scale to Track Entry and Differentiation of myeloid cells in the Allergic Lung

Experimentally induced allergy in mice using ovalbumin (Ova) and house dust mite (HDM) allergens (S1A Fig) has been routinely used to mimic allergen-induced inflammation in human lungs [7,11,18]. These models, like some types of human asthma, are characterized by dramatic increases in lung monocytes and monocyte-derived cells, acetylcholine-induced bronchial hyper-responsiveness, eosinophilia, and increased allergen-specific serum IgG. While perhaps representing only a subset of human allergic lung disease, these pre-clinical mice models provide an important and tractable framework for understanding lung surveillance, antigen sampling and processing and cycles of airway inflammation.

In a previous live-imaging study, we demonstrated that cells expressing a CD11c^YFP^ reporter accumulate around airways in experimentally induced lung allergy in mice, whereas alveolar macrophages (AMs, which can also express this marker under inflammation) remain distributed uniformly throughout the alveoli with no airway accumulation [11]. Adapting that labeling for two-color ratio analysis of the differentiation of CD11c+ cells from their monocyte precursors, we first determined that a CD11c^mCherry^ reporter allele [13] is similarly specific to DC and AM as the CD11c^YFP^ allele and we extended an image-analysis strategy whereby characteristic autofluorescence and sphericity of AM allowed us to differentiate AMs versus DC in lung images and thus observe similar recruitment of CD11c^+^ cells to the airways (S1B–S1D Fig).

To complement this, using a *CX3CR1*-EGFP allele, known to predominantly mark cells of monocyte origin [19], we observed that the allele marks both Ly6C^+^CD11c^-^ monocytes as well as portions of Ly6c^+^CD11c^+^ populations (moDC). In addition, a fraction of the Ly6c^-^CD11c^+^ cells were also positive for the allele, indication that at least some these cells are monocyte-derived (S1E and S1F Fig [see also 7]).

**Fig 1:**
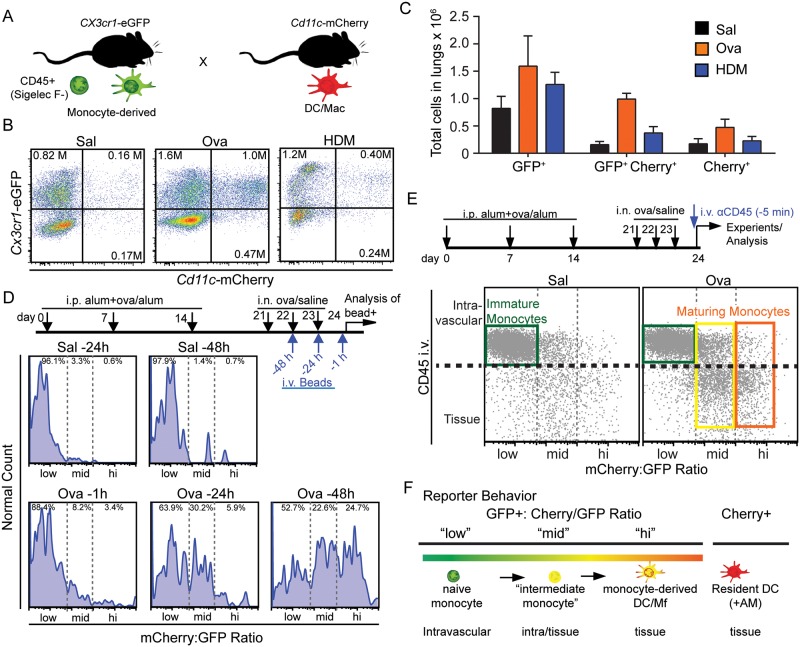
A dual reporter system reveals maturation of monocytes inside and outside of the lung vasculature. (**A**) Illustration of the *Cx3cr1*-eGFP-Cd11c-mCherry transgenic mouse model used for lineage tracing of monocyte to DCs. (**B**) Flow cytometry with absolute numbers (insets) and (**C**) normalized total counts of GFP^+^ and/or Cherry^+^ cells in saline, Ova or HDM-treated lungs within a CD45^+^CD11b^+^Siglec-F^-^Ly6G^-^ gate. Data in C was normalized to input fluorescent polystyrene beads during lung digests. Data in B-C represent 3 independent experiments. (**D**) Experimental layout and results of intravenous labeling of blood monocytes as a pulse with intravenous beads (“pulse”), followed by tracking of bead+ lung-homing monocytes or monocyte-derived cells over 48 hours during the “chase” period. Flow cytometry data is plotted as a ratio of the GFP and mCherry channel with gates for ‘low’ set based on Saline at -24 hours, ‘med’ based on OVA at -24 hours and ‘hi’ representing populations that appear predominantly after 48 hours. Data represent 3 independent experiments. (**E**) Experimental layout and resultant flow cytometric analysis of intravenous labeling of cells with high molecular weight anti-CD45 antibodies 5 minutes prior to euthanasia. CD45^+^CD11b^+^Siglec-F^-^Ly6G^-^ lung monocytic cells are plotted on the identical Cherry:GFP scale as (D), with the CD45 i.v. specifically labeling intravenous immune populations as delineated in S2B Fig. (**F**) Illustration of the extent of monocyte→myeloid maturation and concomitttant tissue distribution that is differentiated by ratiometric dual reporting of *Cx3cr1*-eGFPx*Cd11c-*mCherry mice. Data in D-F representative of at least three mice per condition.

We reasoned that in the intercross cells of the CD11c and *CX3CR1* alleles (Fig 1A), cells of very recent monocyte origin in the lung would be GFP^+^Cherry^-^, whereas monocytes that began to differentiate to express CD11c would then be both green and red (“GFP^+^Cherry^+^”). We further hypothesized that by tracking the absolute intensity of the red signal within the GFP^+^ populations, we might generate a more granular measure of maturity, wherein the ratio of red and green measure the time and/or degree of maturation of the monocytes. To study this, we first performed flow cytometry of dissociated lungs to ascertain, in this mouse, which populations were enhanced under allergy. This demonstrated that Ova or HDM models for allergy both resulted in an increased number of monocyte-origin GFP^+^ and GFP^+^Cherry^+^ as well as likely monocyte-independent Cherry^+^ (only) cells within the lungs, with the maximal increase (>5 fold) being in the GFP^+^Cherry^+^ population (Fig 1B and 1C).

Then, to test whether this marker combination provided a robust measure of differentiation, we performed a pulse-chase labeling of blood monocytes and tracked them and their offspring into the colored populations. We performed i.v. injection of polystyrene beads which specifically labeled precursor peripheral blood monocytes [20]. We did this at different time points during Ova challenge and then measured the bead^+^ cells in GFP and Cherry channels by flow cytometry at later times as they ‘chased’ into the lung. Data was then expressed as a calculated ratiometric channel, derived from the single channel measurements. We partitioned this data into three ratio ‘levels’ representing three prominent differentiation states (Fig 1D). In control mice, bead-labeled offspring remained at the low end of a Cherry:GFP ratiometric scale over 48 hours when assessed in saline (control) challenged mice (ratio “low”). In allergic mice, the initially green-only bead^+^CD11c^-^ monocytes developed within 24 hours into GFP^+^Cherry^+^ (bead^+^) moDC/MΦs that were towards the middle of the ratiometric color scale (ratio “mid”). And, 48 hours later we observed greater numbers (~25% of total bead+ cells) of *CX3CR1*^lo^CD11c^+^bead^+^ mature DCs that were towards the high end of the ratio scale (ratio “hi”). Injected beads resulted in less than 1% of labeling within the Cherry-only (“Cherry^+^”) cells consistent with those cells being largely tissue resident and not effectively surveying the blood (S2A Fig). Since free beads did not persist in the blood (data not shown), this pulse-chase method demonstrates that maturing and mature GFP^+^Cherry^+^ leukocytes in allergic lungs are derived from peripheral-blood GFP^+^ monocytes within 48 hours of the recall response, and broadly demonstrates movement from the lower to the higher end of the Cherry:GFP ratiometric scale as differentiation proceeds.

We next sought to determine whether the ratiometric scale would rise in correspondence to the movement of monocytes from within the vasculature to within the parenchyma. To this end, we adopted a vital pulse-labeling strategy [14,21] to label intravascular and marginating leukocytes. This method uses intravenous injection of an anti-CD45 antibody conjugated to a bulky APC moiety that prevents the antibody from easily leaving the circulation (Fig 1E) followed quickly by tissue digestion and flow cytometric analysis of populations for the presence of the vascular mark. The validity of this method was confirmed by the absence of staining in AM and eosinophils (only in allergy) which are both known to reside abluminal to blood vessels and by the strong antibody staining of neutrophils which reside largely within vessels at the steady state (S2B Fig).

Using this method, the lowest Cherry:GFP ratios, defined identically in Fig 1D, stained brightly with anti-CD45 indicating intravascular localization (Fig 1E), whereas the freshly-homed CD45^-^ cells were found only amongst the ratio-mid or ratio-high populations. This suggests that they had already begun to differentiate during or quickly following extravasation. Additionally, when monocytes and their offspring were assessed, we found that ratio-low cells were almost exclusively CD45 i.v. bright, that ratio-mid cells were mixed with some labeling brightly but the majority being CD45 i.v. low. Ratio-hi cells were almost exclusively CD45 i.v. negative. We confirmed, however, that in the lungs, 10–15% of cells gated by Ly6c-CD11c+ also showed weak staining for CD45 (S2B Fig). This observation may be attributed to either some interstitial DCs gaining access to vessel contents, or *in vivo* CD45 labeling of previously characterized Ly6C- monocytes [3]. From the sum of this data, we concluded that the ratio scale defined by these two markers delineates intravascular monocytes at the lower end, and it shows that early differentiation (ratio-mid) cells are split between a minority that are intravascular and a majority that are within tissues, while confirming that ratio-hi (and indeed monocyte-independent Cherry-only S2A Fig) cells are nearly all within the tissue.

To complement our findings to known markers of monocytes and cells of myeloid origin in the lungs, we took advantage of FcγR1^+^/MerTK^+^ [7,8,22] labeling which has been suggested to define mature/maturing MΦ but not monocytes or DC and found that virtually none of the GFP^+^only cells expressed these markers but that from ~53% of the GFP^+^Cherry^+^ cells did so in control mice and ~17% in allergic mice (S3A and S3B Fig). Only a very small percentage, typically <2% of the Cherry^+^only population were positive, consistent with this marker selecting against those that would be considered ‘DC’. While the change from 53% to 17% indicates a potential bias against ‘conventional macrophages’ in models such as Ova-induced lung allergy, the bulk of this data helps to confirm that the ratio-lo population predominantly represents monocytes, whereas the ratio-mid and ratio-hi (collectively GFP+Cherry+ in this gating) cells contain differentiating cell populations and finally that the Cherry-only cells are largely not macrophages by this definition[7].

### Monocytes and moDCs are all enriched near airways within allergic lungs

**Fig 2:**
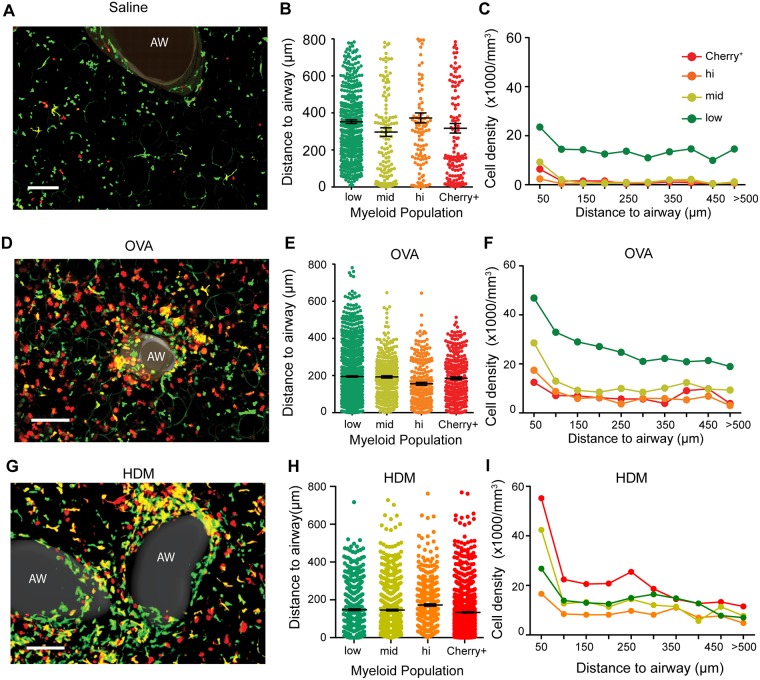
Airway-proximal accumulation of myeloid populations in inflamed mouse lungs. Mouse lungs were sectioned and analyzed using the color labeling strategy defined in Fig 1. Bins were set similarly to flow, using the transformation shown in S3 Fig. (**A,D,G**) Two-photon micrographs of control (A) Ova- (D) or HDM (G) treated lungs from *Cx3cr1*-eGFP x *Cd11c-*mCherry mice. Scale bar = 100 μm. ‘AW’ = airway. (**B,E,H**) Distances of myeloid cells from nearest airway epithelium in control (B) ova- (E) or HDM (H) treated lungs. Myeloid populations were categorized into divisions based on the imaging Cherry:GFP color scale, as described in S3 Fig. (**C,F,I**) Density of myeloid populations in control (C) ova- (F) or HDM (I) treated lungs, as a function of distances from airways, using the identical ratio color scale as in panels B,E,H. See also Video 1. Data represent 4 independent experiments for each condition.

Having established a basic schema whereby a two-color marking scheme could differentiate vascular-resident monocytes, recent moDC/MΦ and resident DC, we next employed laser scanning multiphoton and confocal microscopy of vital lung sections [11] to observe the spatiotemporal dynamics of these populations within the distinct niches of the lungs parenchyma. The use of lung sections, imaged at 30” intervals for 20–30 minutes of total time was used in place of intravital imaging since the latter did not reach the medium and large airways where immune accumulations are most prominent in mouse and human lung allergy [17]. Lungs that had been extensively perfused with saline, nevertheless exhibit significant numbers of undifferentiated GFP^+^only monocytes, ‘marginating’ in vasculature and comparatively few double-positives and Cherry-only cells (Fig 2A). Through quantification of hundreds of cells over multiple imaging fields and using gates that were matched quantitatively to those established in Fig 1 by flow (S3C Fig), we observed that ratio-lo, ratio-mid and ratio-hi populations were all, on average, located closer to airways under allergic challenge (~200μm average distance Fig 2E and 2H) as compared to in control conditions (~300–400μm average; Fig 2B). Additionally, whereas all subsets were somewhat evenly spaced in control lungs (i.e. the density versus distance relationship was flat, with the exception of a slight enrichment in the first 50 μm adjacent to airways, Fig 2C), monocytes (ratio-low) and especially ratio-mid and ratio-hi moDC/MΦ demonstrated a slope for density versus distance following antigen challenge (Fig 2F–2I). This result shows that, already prior to their extensive differentiation, that monocytes are enriched near airways. Additionally, that the enrichment tails off for all populations over ~300 μm is consistent with a chemotactic gradient as opposed to simple trapping in specialized airway-adjacent parenchyma.

### Differentiation State-specific Changes in Morphology and Motility

**Fig 3:**
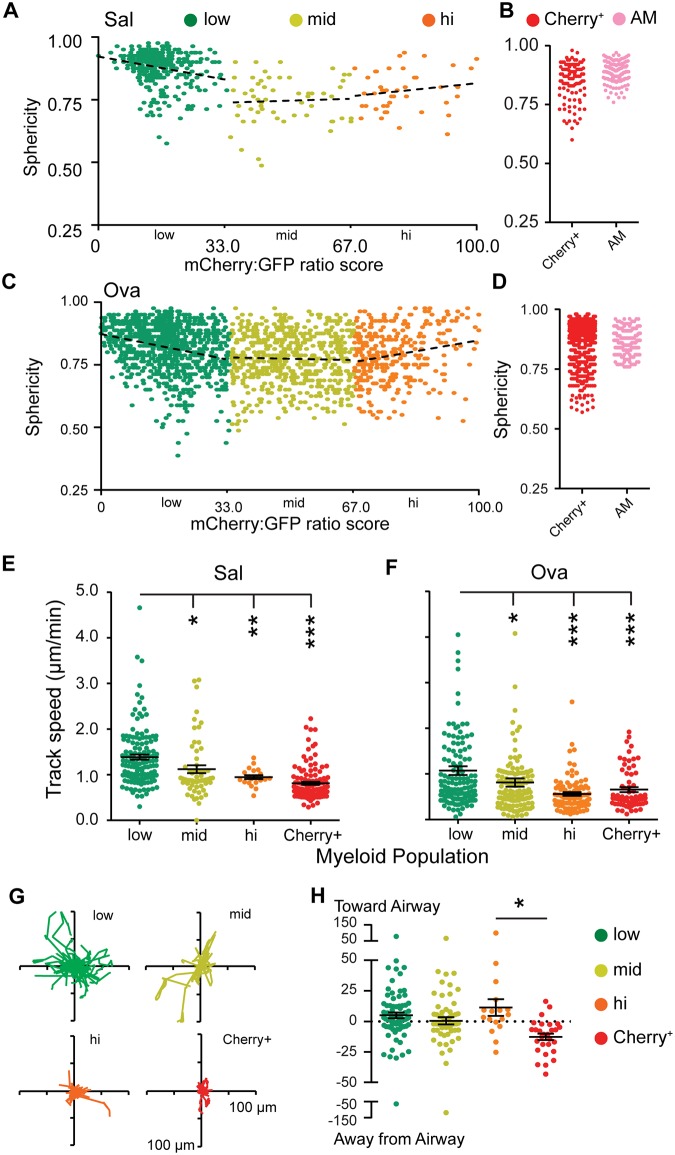
Increased sphericity and reduced motility of monocyte-derived populations as they differentiate in inflamed mouse lungs. (**A,C**) Sphericity of GFP^+^ leukocytes in control (A) or Ova-treated (B) lungs categorized into divisions based on Cherry:GFP ratio score or Cherry^+^only cells and AMs. (**B,D**) Normalized trajectories of cells in panels A and B respectively (Also see S1 Video). (**E,F**) Track speed mean of cells in panels A and B respectively. * *p*<0.05; ** *p*<0.005; *** *p*<0.001. (Pairwise t-test compared to ratio low cells). (**G**) “Flower” displacement plots of 20 randomly selected tracks from each differentiation bin. (**H**) Track displacements of cells from panel A with respect to the airway. Positive numbers indicate movement towards, and negative numbers away from the airway. * *p*<0.05. All others are pairwise not significant at the 95% confidence interval (Unpaired t-test). Data in **A-H** are from 4 independent experiments.

We next analyzed morphology and motility parameters for populations across the ratiometric differentiation color scale. We quantified sphericity (the degree to which cells are round) and found that both in control (Fig 3A) and in Ova-challenged (Fig 3C) mice, ratio-low cells were typically round, whereas maturing ratio-mid and ratio-hi moDC/MΦ typically became more elongated, showing evidence of multiple long protrusions resembling dendrites. This shift may in part be a result of maturation itself or might also be in part influenced by the topology of the interstitium of the lung which itself is tortuous. Cherry-only (Fig 3B and 3D; typically not recent emigrants) were similar in sphericity to the ratio-hi while AMs displayed high sphericity as part of their definition—which has been previously confirmed by Siglec-F staining[11].

We next used timelapse imaging (S1 Video) to measure motility parameters for each of the populations. Mean speeds (Fig 3E and 3F), Flower plots of motility (Fig 3G) and timelapse videos (S1 Video) all demonstrate that the ratio-low (monocytes, intravascular, Fig 1) move faster and displace more significantly, relative to the later differentiation states. For the ratio-low monocytes, we found average speeds were greater than 1 μm/min. Comparatively, ratio-mid and ratio-hi cells typically moved at speeds of <1 μm/min and this was observed in both control (Sal) and Ova-challenged lungs (Fig 3E and 3F). Maturing cells also displaced much less widely (Fig 3G, shown for Ova). However, while such flower plots demonstrate the total rate of displacement over time, analysis of the average directionality of the total track displacements relative to airways showed that ratio-low, med and high vectors had slight biases relative to airways demonstrating slow but net movement towards airways (Fig 3H, shown again for Ova-challenge). In contrast, Cherry^+^only cDC derived cells typically moved modestly away from the airway, a result which we speculate may be due to their entry into or movement within lymphatics, toward lymph nodes. It is possible and indeed likely that all motion in the interstitium might be accelerated in the fully functioning lung as a result of lymphatic flow and tissue movements. But at this time, intravital imaging is limited to approximately the first 2 alveoli in distance from the lung pleura[17], therefore we can only visualize motion that is cell-intrinsic and that accesses tissue biology (e.g. persistent chemokine gradients) that are maintained in a lung slice culture. It thus remains an open question as to whether additional changes in motility would be observed for these myeloid populations under fully intact physiology.

**Fig 4:**
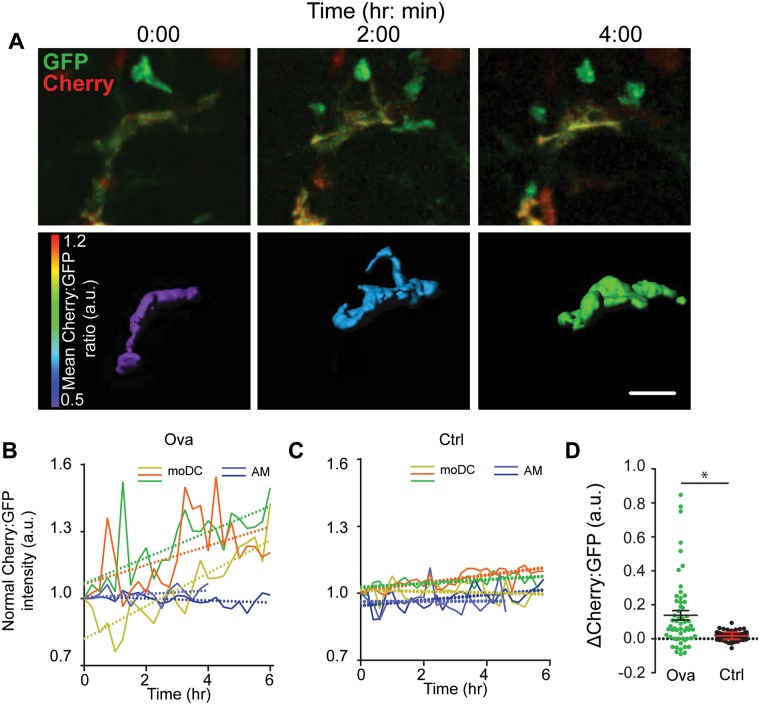
*In situ* maturation of *CX3CR1*^+^ monocyte-derived cells within inflamed lungs. (**A**) Two-photon micrographs at three selected time points demonstrating in situ upregulation of the mCherry signal and thus advancement in red/green ratio. Top row shows an extended myeloid cell, showing Cherry (red) and GFP (green) channels. Bottom row shows rendered isosurfaces of the same cells, using a Cherry:GFP ratio-based color scale. Note: 0h is the beginning of imaging on day 24 of ova treatment. Scale bar = 20 μm. Also see Video 2. (**B**) Cherry:GFP ratio, normalized to the first frame, for 3 exemplar moDCs along with similar plots for 2 alveolar macrophages (AM) for comparison. Linear fits are shown by dotted lines, Each color represents an individual cell. (**C)** Similar plots for three cells taken from control lungs, also compared to exemplar AMs and showing reduced in situ maturation. (**D**) Plots of change in Cherry:GFP ratio normalized to AM intensity and time, for multiple cells studied in OVA-challenged and control lungs showing statistically significant p<0.05 differences in allergic lungs as compared to control. Data represent 3 independent experiments.

We next asked whether we could track the ratio via imaging at longer frame-intervals (10–30 minutes) and over 8–10 hour imaging sessions in order to document differentiation *in situ*. We found that we could indeed observe cells that were up-regulating Cherry within the GFP^+^ cells in allergen-challenged lungs, over this time course. One example of this is shown in Fig 4A/S2 Video. Here, a slowly migrating cell with elongated morphology can be seen transitioning from a greenish to a more yellow cell (top) and a ratio “mid” to a ratio “hi” state (below) over 4 hours of imaging. To quantify these types of transitions over multiple cells under allergy or control conditions, we plotted the ratio of three such cells, normalizing each at a ratio value of ‘1’ at the beginning of the observation (Fig 4B and 4C). This demonstrated GFP+ cells with upward slopes in the ratio under Ova allergen challenge (Fig 4B). To analyze the effects of bleaching and as a control, we assessed the ratio for AMs, which are Cherry^hi^ but only express autofluorescence in the GFP channel and for monocytes which are GFP^+^ but Cherry negative (data not shown). These demonstrated that such a rise in ratio was specific to cells with intermediate ratios and was not a consequence of differential bleaching of one fluorophore compared to the others. Most importantly, most monocytes we studied showed evidence for in situ differentiation on the time-frame of hours in allergen challenge conditions but no such changes in control lungs (Fig 4C and 4D). This indicates that, while maturing monocytes do exist in normal lungs, that their rate of maturation is enhanced alongside the rate of recruitment, under inflammatory conditions.

### Convergent improvements in Antigen Capture and Induction of T cell arrest, for Maturing Monocytes

We next sought to understand how these differentiation states related to the functional roles of monocyte-derived and resident myeloid cells as antigen presenting cells *in situ*. While both of these parameters has been demonstrated to improve in vitro, it is more difficult to study in vivo and no direct comparisons of this sort have, to our knowledge, been demonstrated. Our ratio system now gave us the opportunity to analyze antigen uptake across differentiation states and we initially turned to the physiologically-relevant HDM-inhalation model wherein the particulate is a complex material, highly associated with human asthma.

**Fig 5:**
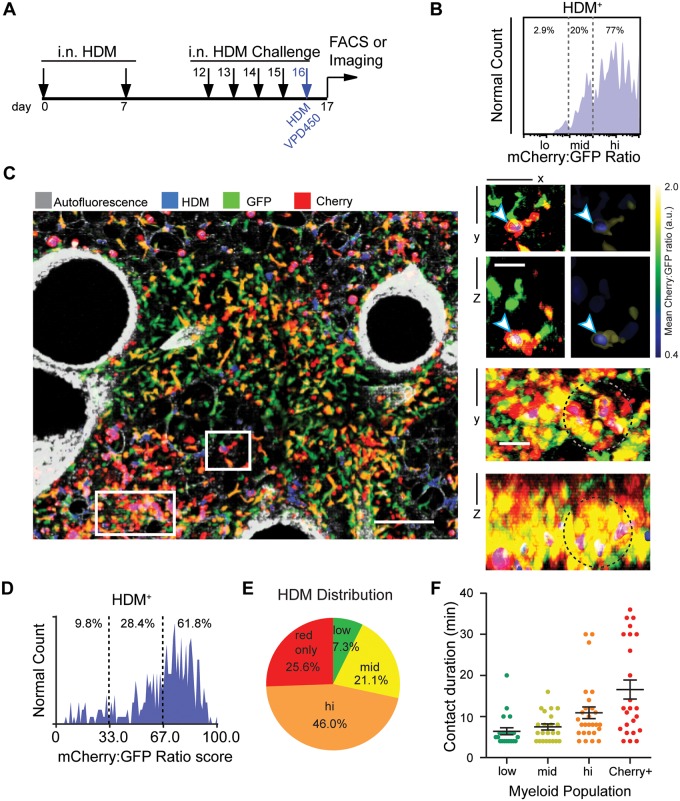
Antigen uptake and T cell engagement increase for differentiated monocytes. (**A**) Protocol for inhalation of fluorescent VPD450^+^ HDM to score for efficiency of antigen capture in myeloid cells *in situ*. Labeled HDM was administered i.n. on day 16 in a protocol which otherwise closely resembled that outlined in S1 Fig. (**B**) Flow histogram showing distribution of HDM taken up by CD45^+^CD11b^+^Siglec-F^-^Ly6G^-^ lung leukocytes on the Cherry:GFP color scale, with percentages of VPD450+ cells shown above and demonstrating dominance of uptake by the most differentiated cells. (**C**) Two photon micrograph of HDM-treated lungs showing GFP^+^ and/or Cherry^+^ cells. Note blue positivity for multiple populations and across the lung. Scale bar = 100 μm. Right: XY and XZ sectional views of the regions marked in C showing HDM particles within moDCs (white arrow, bound by blue) and accumulation of ratio-mid and ratio-hi cells around HDM particles (beads appear purple, region of interest indicated by dotted circles). Scale bars = 20 μm. (**D, E**) Quantification of endocytosed HDM observed by two-photon microscopy, shown as **(D,** histogram; excluding Cherry^+^only cells) or including (**E**, pie chart; including Cherry+only cells). (**F)** Contact duration between adoptively transferred and labeled OTII T cells and the indicated myeloid population at 16 hour post T cell transfer in a similar OVA-model for antigen uptake (see S4 Fig), derived from timelapse imaging of lungs as previously described[11]. Dwell times > 4 min are considered. All data taken from at least 3 experiments.

To track uptake in the dual-marked mice, animals sensitized with HDM received their final challenge of fluorescently-labeled HDM on the penultimate day of the regime (Fig 5A). Flow cytometry, gating on the HDM positive population within the GFP (recent monocyte-derived) populations, revealed that cell-associated fluorescent HDM was found predominantly in cells at the the mid (22%) and hi (77%) range of the color scale (Fig 5B), also consistent with the observation (Fig 1) that the ratio-low population was mostly intravascular and so might not efficiently sample the airways.

Using multiphoton fluorescence microscopy we observed that HDM particles were physically close to cells within the entire ratio range (Fig 5C). As observed by FACS, we rarely observed ratio-low monocytes containing intracellular HDM by imaging, although we frequently found these cells within less than a micron of HDM fluorescence in the parenchyma (Fig 5C, insets). This lack of phagocytosis may be attributed to either insufficient material being passed the across the endothelium and epithelium from the larger HDM granules, or, even if the monocytes do get access to the alveolar mucosa [3], they may be unable to phagocytose sufficient material from the large HDM granules for us to detect. In contrast, we frequently observed ratio-mid and ratio-hi cells around the alveoli that had at least partially ingested an HDM particle (Fig 5C, insets).

We quantified this data from imaging, first establishing surfaces on each cell and then asking whether the volume bounded by those regions contained blue fluorescence. We found good concordance with the flow-based study (Fig 5D); imaging documented 9% of HDM associated with ratio low whereas flow cytometry estimated that at 2% and flow cytometry showed that ratio-hi cells captured 77% of HDM whereas imaging measured this at 62%. Differences in these values may reflect small regional effects for the imaging, cell isolation from tissue, or slight variations in cutoffs between ratio levels in the two techniques. As a comparison, we also measured uptake by the less –populous mCherry-only (non-AM) population and found when they were included, they were responsible for approximately 26% of all phagocytosis, suggesting that they were at least as able, if not more so, to access an internalize HDM (Fig 5E**)**.

A large fraction of human asthma cases, and indeed both of the mouse models used here, are characterized by a Th2-mediated immune response. We have previously demonstrated that Th2 interactions with intra-lung CD11c+ occur as “swarms” adjacent to medium/large airways and involving transient calcium signaling by responding T cells [11]. To study the ability of the various differentiation states to capture T cells in T-APC contacts in vivo, we took advantage of the Ova-allergen model for which a readily available TCR transgene, OTII, can be introduced to measure T cell behavior. We first studied the uptake of OVA-fluorescent beads as surrogates for particulate antigen and found that, like for particulate HDM, accumulation of these was again biased towards the more differentiated ratio-mid and especially ratio-hi populations (S4A–S4C Fig).

Next, when pre-activated antigen-specific T cells were introduced into our color-marked mice, we found that with 16h, they clustered in the airway-proximal region (see also [11]) and engaged in interactions with our labeled populations there. The duration of T cell interaction there followed maturation state; cherry-only cells generated the longest interactions, followed by ratio-hi, ratio mid and finally ratio-low (Fig 5F). XCR1 has been previously shown to distinguish conventional CD103^+^CD11b^hi^ airway DCs [23] that are also known to express CD26 from moDCs [24,25]. We utilized vital XCR1 staining to determine the presence of conventional DCs within our differentiation color-scale. Results showed that while ~15% of Cherry only cells are also XCR1+, the proportion of XCR1+ cells is low (~2.8%) in “hi” population, and close to undetectable in the “mid” and “low” populations (S4D Fig). These results indicate that both conventional as well as moDCs participate in antigen-specific T cell-APC interactions in inflamed lungs.

## Discussion

Using a real-time method that measures monocyte maturation, behavior and function simultaneously and directly in tissue, we have demonstrated spatial and functional accumulation of antigen-presenting cells near proximal airways. Our data suggests a preferential homing of monocytes near airways but also shows that maturing monocytes migrate slowly as they continue to differentiate. Given such slow directional migration in ratio-mid and ratio-hi moDC/MΦ, we may now suggest that ‘focusing’ of immune response to airways is both initial recruitment of monocytes as well as likely a consequence of continued migration of partially differentiated cells. Additionally, this work provides important insight on the functional analysis of undifferentiated or partially-differentiated cells of monocytic origin, suggesting that these cells really are poor antigen-presenting populations in their undifferentiated or semi-differentiated state, but are effective in T cell engagement upon complete differentiation.

The overall motility of cells studied here is likely under-representative of motion in a fully intact lung. Prior to this work we appreciated, from similar organ imaging of Rodero et al. [3] and similar imaging in mesenteric vessels [26], that monocytes patrol the vasculature in the steady state. In addition, high-motility patrolling of cFMS-EGFP bearing cells, which includes monocytes was observed in intravital lung imaging in a previous report [17]. In a lung-slice preparation as we studied here and by Rodero et al. [3], it is particularly apparent that such motility absolutely does not rely on blood flow but is instead an intrinsic property of the monocytes themselves. At present, we suggest that motility rates in slice, as ours, underestimate the actual rates as blood flow (for intravascular populations) and normal lung expansion/contractions (for interstitial populations) will generate additional forces that will move these cells. Fully intact lungs may also contain additional chemokine gradients that are lost during our slice-preparation, further underestimating motility.

Such shortcomings notwithstanding, this work defines multiple states of monocyte differentiation, with respect to spatial localization and defines how these cells change with each step of differentiation. Recent imaging of Rodero et al, similar to ours but using single channel reporting of monocytes and macrophages [3], already showed clear differences the motility of vascular CX3CR1+ monocytes cells as compared the broad population of interstitial CX3CR1+ populations that include monocytes at varying degree of maturation. Prior to our imaging work, Jakubzick et al have shown that the lungs contain ‘partially differentiated’ monocytes and that they or their early progeny are capable of some antigen uptake and processing [8]. Here, we have been able to both localize such partially-differentiated cells, namely the ‘mid ratio’ cells in our study in context, finding that they appear relatively stuck in that differentiation state in normal lungs (e.g. Fig 4C and 4D). Additionally, we show that, though resembling their more mature cousins for distribution, morphology and motility, that their capacity both as antigen-capture and as mediators of T cell activation is quantitatively weaker *in situ*. Once they further differentiate, the ratio-hi cells that we describe are almost certainly similar to what is also referred to as moDC and have been previously implicated as major mediators of mouse lung allergy in previous studies [7]. Our results in this regard are relatively in concordance; ratio-hi cells are the most abundant population in active asthma, accumulate approximately twice as much HDM as any other single population (Fig 5E) and mediate significant T cell interaction (Fig 5F). To this extent, our work also extends the findings of Rodero et al, using the imaging approach to further differentiate monocyte-derived populations in situ [3].

Based on the data we’ve collected there remains finer details to be resolved. Namely whether there is selectivity for undifferentiated monocytes cells to migrate directionally toward airways (suggested in Figs 2 and 4) or a selectivity for cells that are moving near/towards airways to differentiate. Additional testing of these options will require development of significant new technologies to permit more robust analysis in fully intravital imaging. Regardless, the data suggests a differential focusing of differentiated monocytes with antigens and T cell stimulatory capacity to the proximal airways.

The implications of the finding presented here are that strategies to prevent mono-to-DC conversion or prevention of preferential zoning of these leukocytes by intranasal administration of drugs may represent an alternative and effective treatment for asthma as the end-product monocyte-derived ratio-hi/moDC cells from our analysis perform nearly as efficiently in engulfing allergens and engaging T cells as the resident DC pool. Our work would suggest that, while monocytes with intermediate degrees of maturation toward DC phenotypes are present within the lung, their stimulatory capacity likely requires further maturation signals, perhaps delivered by pathogens or damage cues. Ratio-mid and ratio-hi cells expressed similar levels of MHCII (data not shown) but other factors may prove important. Indeed, this work should be seen as very complementary to recent genetic knockout experiments which suggest that monocyte-derived DC ultimately ‘drive’ allergic responses in HDM models [7]. However, our data would divide such populations into subpopulations and extends such genetic results to precisely spatially and temporally delineate the logic of the maturation of these cells and the measurement of specific functional deficits for the maturing populations. Future therapeutic efforts may focus, therefore, not only on addressing monocyte recruitment per se but also intra-tissue differentiation mechanisms.

## Supporting Information

S1 Fig Lung inflammation models used in this study, methods for excluding AMs based on sphericity and *CX3CR1*-EGFP/CD11c-mCherry intensity, and robust *CX3CR1*-EGFP expression in myeloid cells in the lungs. (**A**) Protocols used for induction of lung allergy in mice using ovalbumin (ova) or house dust mite extract (HDM). i.p.–intraperitoneal; i.n.–intranasal. Modifications to introduce specific fluorophores at different time points during asthma induction are indicated in respective figures.
(**B**) Gating of AMs by normalized autofluorescence and sphericity. Data from two-photon micrographs analyzed using Imaris, based on the high basal (“autofluorescence”) of alveolar macrophages. Signal from a 450/50 band pass emission path is used for autofluorescence measurement, expressed as a ratio with GFP and used together with sphericity measurements to identify alveolar macrophages. AMs defined this way were demonstrably Siglec-F positive (data not shown, see also equivalent data in [11]).
(**C**) Two photon micrographs of saline or ova-treated lungs showing distribution of mCherry-CD11c^+^ cells. AW = airways. Scale bar = 100 μm. Data represent at least 4 independent experiments.
(**D**) Histogram showing density of distribution at varying distances from airways, of CD11c+ APCs or AMs from the micrographs in **C**, separated by sphericity (AM = sphericity > 0.8).
(**E**) Flow cytometry gating strategy used for delineating Eosinophils (EOS), Alveolar macrophages (AM), Neutrophils (Neutro), Ly6C^+^CD11c^-^, Ly6C^+^CD11c^+^ and Ly6C^-^CD11c^+^ cells.
(**F**) *CX3CR1*-EGFP expression amongst cells as gated in **E**. demonstrating profound GFP expression in multiple Ly6C+ and Ly6C- populations. Data represent at least 4 independent experiments.
(PDF)

S2 Fig CD11c-mCherry^+^ *CX3CR1*-EGFP^-^ Cells only minimally load with intravenous beads and i.v. CD45 provides a robust measure of tissue localization. (**A**) Flow cytometry of Cherry^+^only cells in control or OVA-challenged mice, showing minimal i.v. bead positivity in this compartment. Data represent 3 independent experiments.
(**B**) Histograms of anti-CD45 intravenous pulse labeled immune populations in saline or ova-treated lungs. Data was gated for the indicated immune populations as described in S1C Fig. Data represent 3 independent experiments.
(PDF)

S3 Fig Proportions of MerTK^+^ Macrophages within specific monocyte-derived populations in normal and inflamed lungs and the delineation of maturing-populations using a red/green ratio via imaging. (**A**) FACS plots showing expression of CD64 and MerTK in CD45^+^CD11b^+^Siglec-F^-^Ly6G^-^cells in saline or Ova-treated lungs, and
(**B**) relative abundance of MerTK^+^CD64^+^ Macs in GFP^+^ and/or mCherry^+^ populations. Data in A and B represent 3 independent experiments.
(**C**) Schematic representation of “low”, “mid” and “hi” populations from two-photon intensities of GFP^+^ cells in CX3CR1-GFP x CD11c-mCherry mice. Using this method, the normalized ratios on a log scale are divided into three bins at a similar frequency to that produced by the equivalent FACS quantification and processing.
(PDF)

S4 Fig Distribution of monocytes and monocyte-derived cells in HDM-treated lungs, i.n. bead uptake amongst monocytes and monocyte-derived cells. Mice were infused through the intranasal route (i.n.) with OVA fluorescent beads as previously described.
(**A**) Sphericity of total (gray) and i.n. Bead^+^ (blue) leukocytes in ova-treated lungs on a Cherry:GFP color scale.
(**B**) Two photon micrograph of ova-treated lungs showing i.n. Bead uptake in GFP^+^ and/or Cherry^+^ cells. Scale bar = 50 μm.
(**C**) Two photon micrograph (left) and superposed pseudocolored surface showing i.v. beads after 48 h inside a ratio ‘hi’ moDC. Scale bar = 10 μm.
(D) Proportions (indicated in red) of XCR1+ conventional DCs within ‘lo’, ‘mid’, ‘hi’, and Cherry-only (without AMs) populations in ova-treated lungs. Data in A-D represent 3 independent experiments.
(PDF)

S1 Video Multiphoton time-lapse video of monocytes and iDCs in asthmatic CX3CR1^GFP^:Cherry^CD11c^ mice. Video has been represented in a Cherry:GFP ratiometric color scale and corresponds to text Fig 2D. Actual time is indicated in h:mm:ss.
(MOV)

S2 Video Real-time Differentiation of Monocytes in the Lung. Video corresponding to text Fig 3C. Actual time is indicated in h:mm:ss. Surface corresponds to the cell encircled on the left.
(MOV)

S3 Video 3D Two-photon micrograph of moDC carrying i.v. beads 48h post injection. A 3D 90° rotation along X axis showing a bead-bearing moDC. Video progresses from showing actual channel intensities in GFP and Cherry to pseudocolored surfaces towards the end.
(MOV)
