# Supplementary figures and images for: Tracking the Spatial and Functional Gradient of Monocyte-To-Macrophage Differentiation in Inflamed Lung
Source: PLoS One. 2016 Oct 18;11(10):e0165064. doi: 10.1371/journal.pone.0165064 (PMC5068774; doi:10.1371/journal.pone.0165064)

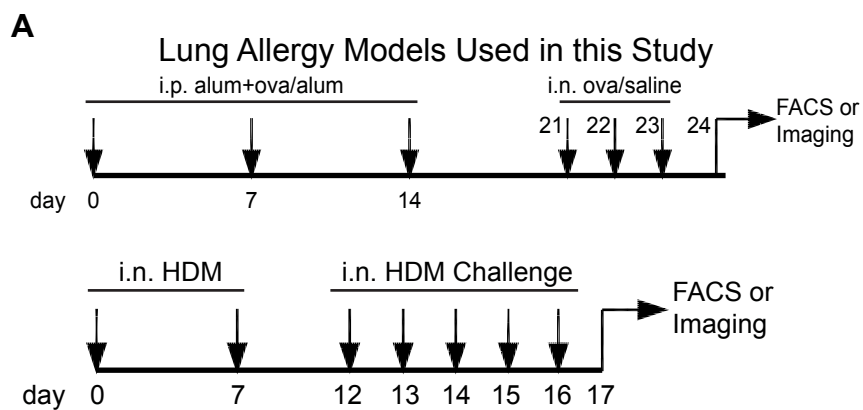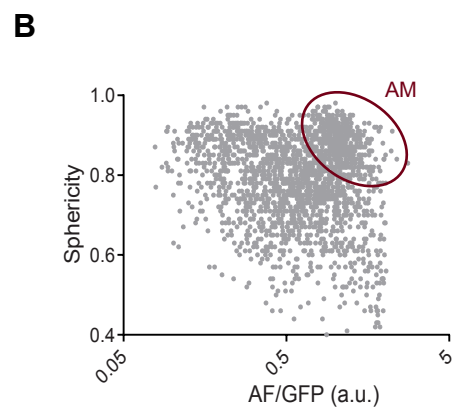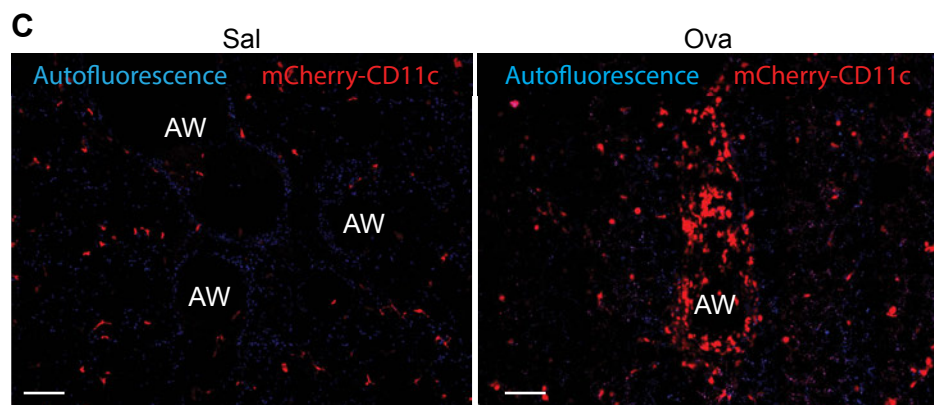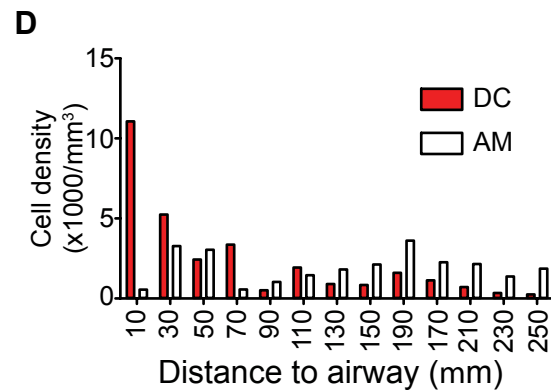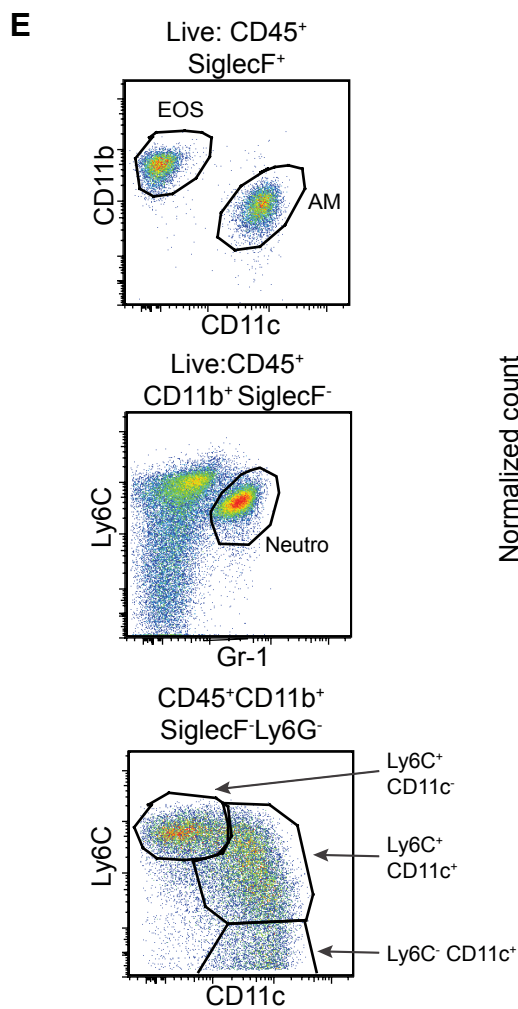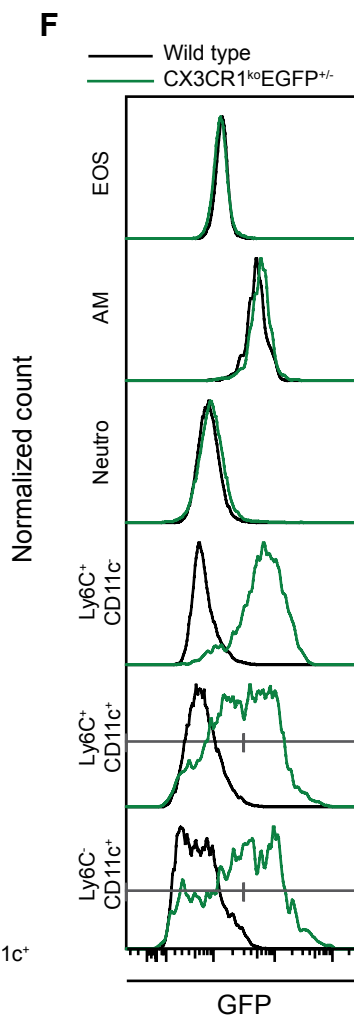

Supplement: S1 Fig — (A) Protocols used for induction of lung allergy in mice using ovalbumin (ova) or house dust mite extract (HDM). i.p.–intraperitoneal; i.n.–intranasal. Modifications to introduce specific fluorophores at different time points during asthma induction are indicated in respective figures. (B) Gating of AMs by normalized autofluorescence and sphericity. Data from two-photon micrographs analyzed using Imaris, based on the high basal (“autofluorescence”) of alveolar macrophages. Signal from a 450/50 band pass emission path is used for autofluorescence measurement, expressed as a ratio with GFP and used together with sphericity measurements to identify alveolar macrophages. AMs defined this way were demonstrably Siglec-F positive (data not shown, see also equivalent data in [11]). (C) Two photon micrographs of saline or ova-treated lungs showing distribution of mCherry-CD11c+ cells. AW = airways. Scale bar = 100 μm. Data represent at least 4 independent experiments. (D) Histogram showing density of distribution at varying distances from airways, of CD11c+ APCs or AMs from the micrographs in C, separated by sphericity (AM = sphericity > 0.8). (E) Flow cytometry gating strategy used for delineating Eosinophils (EOS), Alveolar macrophages (AM), Neutrophils (Neutro), Ly6C+CD11c-, Ly6C+CD11c+ and Ly6C-CD11c+ cells. (F) CX3CR1-EGFP expression amongst cells as gated in E. demonstrating profound GFP expression in multiple Ly6C+ and Ly6C- populations. Data represent at least 4 independent experiments. (PDF) [file pone.0165064.s001.pdf]

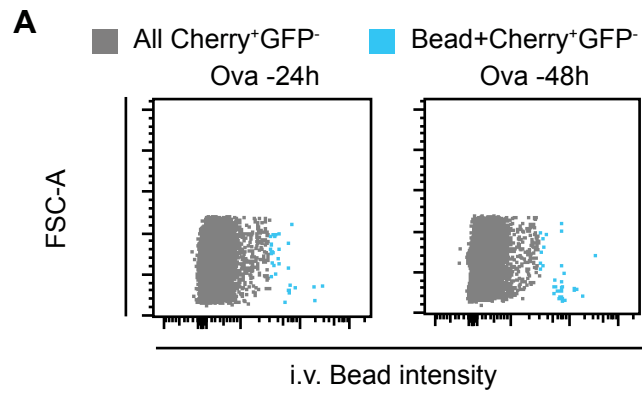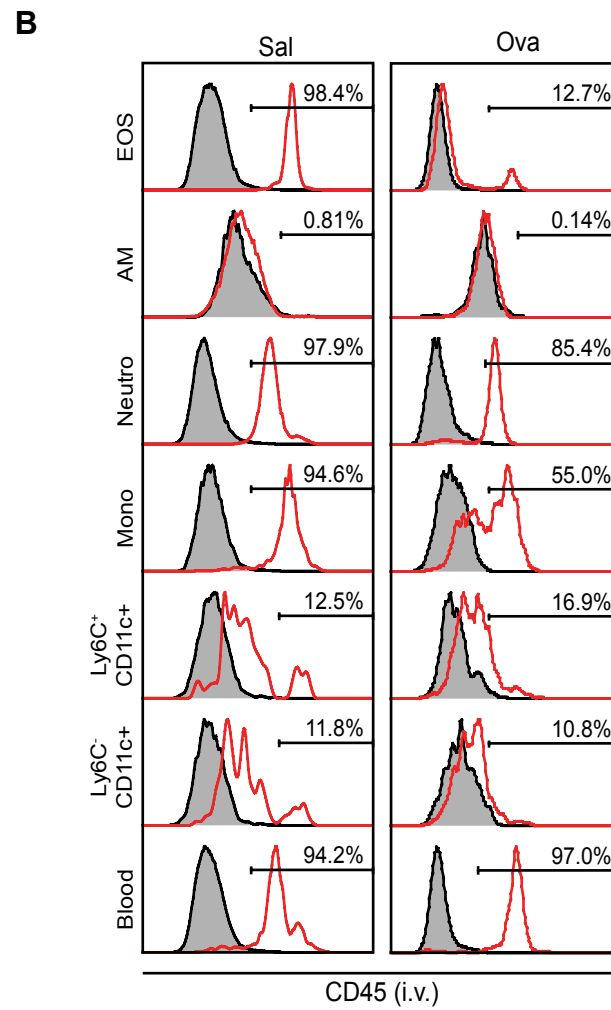

Supplement: S2 Fig — (A) Flow cytometry of Cherry+only cells in control or OVA-challenged mice, showing minimal i.v. bead positivity in this compartment. Data represent 3 independent experiments. (B) Histograms of anti-CD45 intravenous pulse labeled immune populations in saline or ova-treated lungs. Data was gated for the indicated immune populations as described in S1C Fig. Data represent 3 independent experiments. (PDF) [file pone.0165064.s002.pdf]

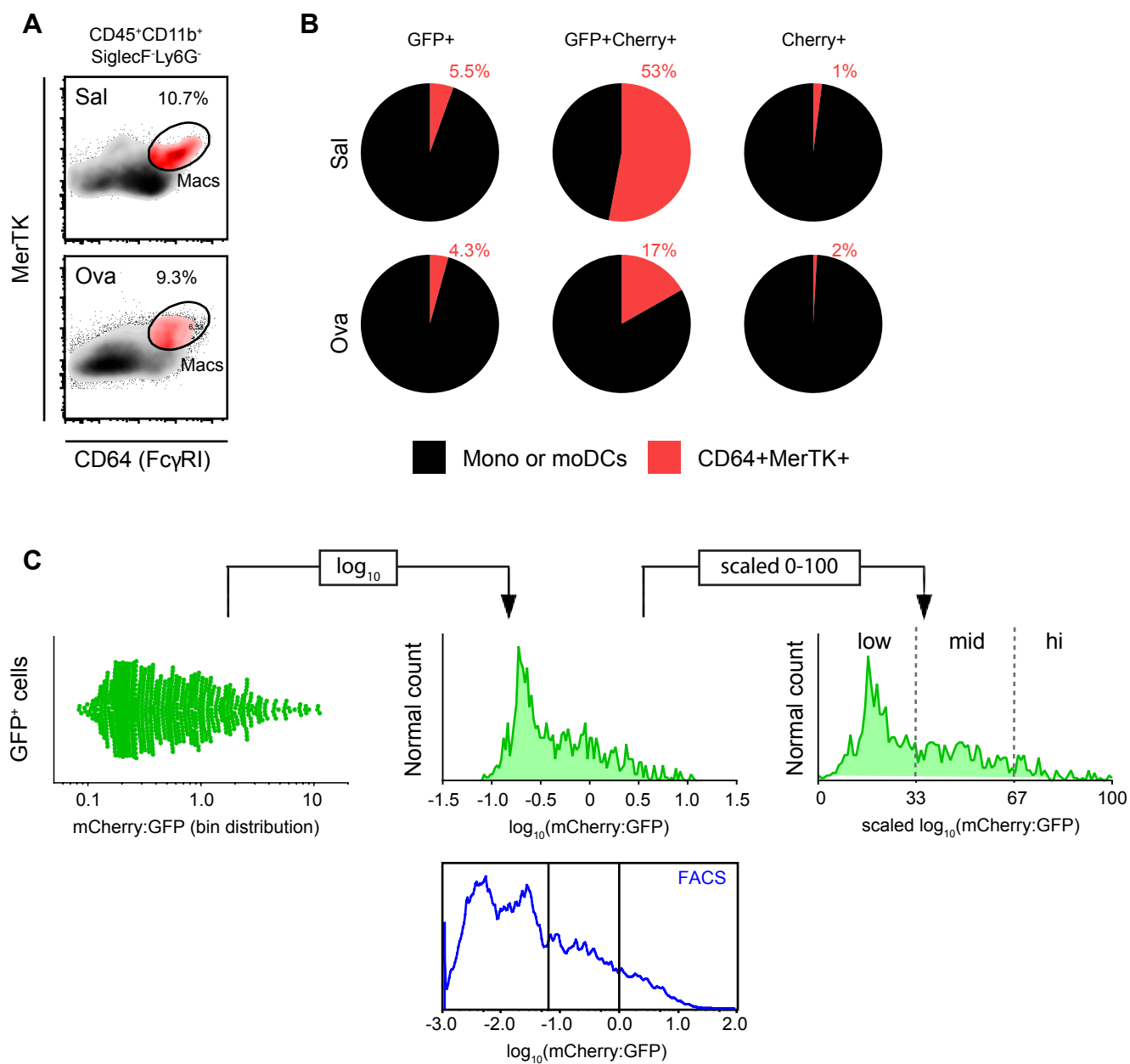

Supplement: S3 Fig — (A) FACS plots showing expression of CD64 and MerTK in CD45+CD11b+Siglec-F-Ly6G-cells in saline or Ova-treated lungs, and (B) relative abundance of MerTK+CD64+ Macs in GFP+ and/or mCherry+ populations. Data in A and B represent 3 independent experiments. (C) Schematic representation of “low”, “mid” and “hi” populations from two-photon intensities of GFP+ cells in CX3CR1-GFP x CD11c-mCherry mice. Using this method, the normalized ratios on a log scale are divided into three bins at a similar frequency to that produced by the equivalent FACS quantification and processing. (PDF) [file pone.0165064.s003.pdf]

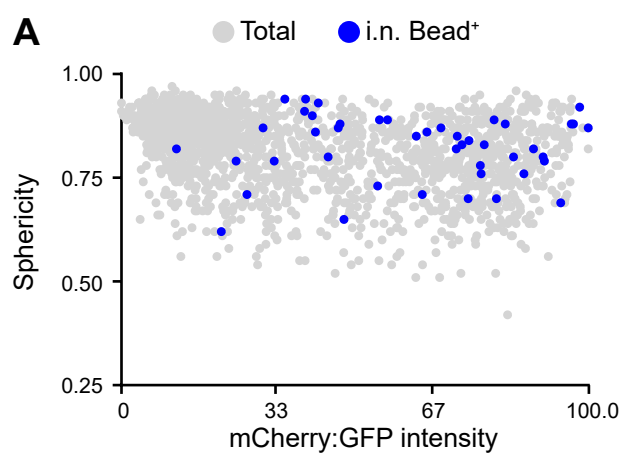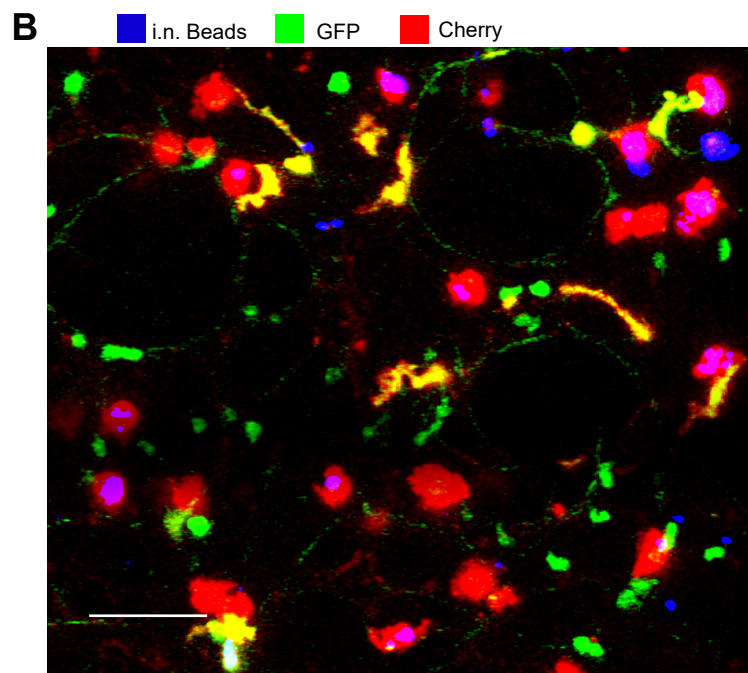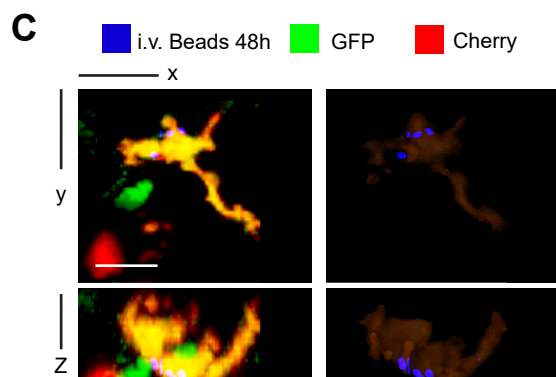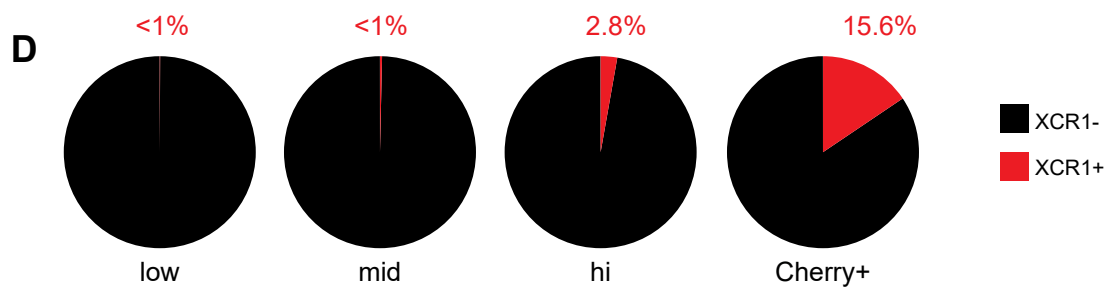

Supplement: S4 Fig — Mice were infused through the intranasal route (i.n.) with OVA fluorescent beads as previously described. (A) Sphericity of total (gray) and i.n. Bead+ (blue) leukocytes in ova-treated lungs on a Cherry:GFP color scale. (B) Two photon micrograph of ova-treated lungs showing i.n. Bead uptake in GFP+ and/or Cherry+ cells. Scale bar = 50 μm. (C) Two photon micrograph (left) and superposed pseudocolored surface showing i.v. beads after 48 h inside a ratio ‘hi’ moDC. Scale bar = 10 μm. (D) Proportions (indicated in red) of XCR1+ conventional DCs within ‘lo’, ‘mid’, ‘hi’, and Cherry-only (without AMs) populations in ova-treated lungs. Data in A-D represent 3 independent experiments. (PDF) [file pone.0165064.s004.pdf]
